# Insight into Oral Biofilm: Primary, Secondary and Residual Caries and Phyto-Challenged Solutions

**DOI:** 10.2174/1874210601711010312

**Published:** 2017-06-30

**Authors:** Smitha Chenicheri, Usha R, Rajesh Ramachandran, Vinoy Thomas, Andrew Wood

**Affiliations:** 1 Department of Microbiology, Karpagam University, Coimbatore, Tamil Nadu, India.; 2 Microbiology Division, Biogenix Research Center for Molecular Biology and Applied Sciences, Thiruvananthapuram, Kerala, India.; 3 Department of Microbiology, PMS Dental college and Research Center, Thiruvananthapuram, Kerala, India.; 4 Department of Materials Science & Engineering, Center for Nanoscale Materials and Biointegration (CNMB), University of Alabama at Birmingham (UAB), Birmingham, Alabama, USA

**Keywords:** Dental caries, Oral biofilm, Phytotherapy, Secondary caries, Transmissible chronic infections, Odontoblasts

## Abstract

**Introduction:**

Dental caries is known to be one of the most widespread, chronic infections affecting all ages and populations worldwide. The plethora of oral microbial population paves way for various endogenous infections and plays a crucial role in polymicrobial interactions contributing to biofilm-mediated diseases like caries and periodontal diseases.

**Methods:**

Extensive literature survey was conducted using the scientific databases like PubMed, Google scholar, Science Direct, *etc*. using the key words like dental caries, orodental infections, dental microbes, dental biofilm, secondary caries, phytotherapy, *etc*. The literature was analyzed thoroughly and critical review was performed.

**Results:**

The risk of development of secondary caries and residual caries further results in treatment failure. Drug resistance developed by oral microbes and further side effects pose serious hurdles in the current therapeutic strategies. The hyperactivities of various MMPs and the resulting massive ECM degradation are the challenging part in the design of effective therapeutic approaches. Anticariogenic phytotherapy is well appreciated owing to lesser side effects and versatility of their action. But appreciable outcomes regarding the phytochemical bioavailability and bioretention are still challenging. Site-specific delivery of phytoagents at the infected site may enhance the efficiency of these drugs. Accordingly emerging phytodentistry can be promising for the management of secondary and residual caries.

**Conclusion:**

This article presents major cariogens and their mechanisms in initiating and aggravating dental caries. Effectiveness of phytotherapy and different mode of action of phytochemicals against cariogens are outlined. The article also raises major concerns and possibilities of phytochemical based therapeutics to be applied in the clinical arena of caries management.

## INTRODUCTION

Dental caries is known to be one of the most rampant and chronic infections of the tooth, globally affecting all ages and populations resulting in severe socioeconomic burden. This multifactorial disease is characterized as a progressive dissolution and demineralization of tooth followed by microbial metabolism. Caries results from complex interactions between tooth structures and oral microbial flora, dental biofilm formation, dietary remnant accumulation, salivary dysfunction and genetic disturbances [1, 2] and progresses by disturbing the equanimity of mineralization. Frequent carbohydrate exposure and/or decreased salivary clearance of food remnants alters the microbial homeostasis of oral cavity which favors biofilm formation leading to caries development [3]. The inhibition of caries development can be achieved by arresting biofilm formation that forms as a result of the onset of microbial colonization [4]. The diagnosis implies not only the detection of caries but also includes the assessment of its status as either arrested or active. If active, further diagnosis is described as whether the progression is rapid or slow.

The mid 20^th^ century witnessed the discovery of etiopathogenesis of cariogenic indigenous microbial commensals of the human oral cavity in dental and periodontal infections [5, 6]. Advancements in molecular biology and genetics have helped to identify more than 800 different species from human oral cavity where a healthy individual may harbor up to 600 species of microbes at any instant of time, still approximately 35% have not yet been cultured *in *vitro** [7, 8]**.** Studies using gonobiotic and conventional animal models have shown that certain endogenous oral bacteria were more virulent than others. Such micro-flora can act either individually or as union of several species through biofilm formation [6, 9-11]. Interestingly, the cariogenic bacteria make up less than 1% of the oral biofilm and include members of oral *streptococci*, mainly **Streptococcus* mutans*, **Streptococcus* sorbinus, *Streptococcus* cricetus, *Streptococcus* rattus, *Streptococcus* salivarius, and *Streptococcus* sanguis*. Other cariogenic bacteria like **Lactobacillus* acidophilis, *Lactobacillus* casei, Actinomyces naeslundii, Actinomyces viscusus*, ***Enterococcus* faecalis*, and *Candida albicans** also contribute to biofilm formation [12]. Under extended acidic conditions, aciduric bacteria dominate along with strains of non-mutans *streptococci*, *Actinomyces*, *Bifidobacteria,* and yeasts. Apart from these initial colonizers, the microbial co-aggregation exists among bacterial species like *Fusobacterium*, *Veillonella*, *Haemophilus*, *Campylobacter, Neisseria, Gemella, Granulicatella, Capnocytophaga,* and *Bacteroides.* These secondary colonizers and their association with tertiary colonizers, comprising of Gram negative anaerobic microbes, play a major role in oro-dental infections [13].

Further caries progression and demineralization of dentin lead to destruction of the collagenous matrix of dentin. Reduction of mineral contents, increases in porosity due to variation in dentine collagen structure and alteration of non-collagenous proteins are other ill effects. These factors significantly decrease mechanical and physical properties of dentine. The host-derived Matrix metalloproteinases (MMPs) play a major role in the degradation of organic matrix of carious dentin. The MMPs can degrade components of ECMs, including fibrillar and non-fibrillar collagens, fibronectin, laminin, and basement membrane glycoproteins [14].

Odontoblasts produce the MMPs in its inactive proforms, in dentine during the secretion of dentin matrix. During mineralization these MMPs along with TIMP remain trapped within the calcified matrix. MMPs get activated upon re-exposure during dentinal caries. The low pH created by bacterial acidic metabolites activates the endogenous MMPs by cleaving the prodomain. Though these MMPs show stability in acidic pH, their functionality is highest at neutral pH, which is facilitated by the salivary or dentinal buffering mechanisms facilitating the degradation of matrix components. Tissue inhibitor of Metalloproteinases (TIMP), the endogenous MMP inhibitors inactivates MMPs and balances the further degradation of the ECM components and thereby plays a crucial role in maintenance of the healthy tissues [15].

MMPs are a family of Zn^2+^- and Ca^2+^-dependent enzymes, able to degrade practically all ECM components hence contributing to significant biological and pathological process. The 23 members of MMP family are frequently divided into six groups collagenases, gelatinases, stromelysins, matrilysins, membrane type MMPs and other MMPs based on the substrate specificity and homology. Proteolytically active MMPs in a carious dentine include collagenases (MMP-1, MMP-8), gelatinases (MMP-2, MMP-9) stromelysin (MMP- 3) and enamelysin (MMP-20). MMP- 8 is most important collagenase effective in hydrolyzing type I collagen fibrils and MMP-9, the predominant gelatinolytic enzyme detected in carious lesions. Apart from this cysteine, cathepsins (B and K) also participate in dentinal caries development. Its activity increases towards depth into the pulp and hence is highly associated with active carious lesion. They have an optimal activity in a slightly acidic pH, generally between 5 and 6. They directly participate in proteolytic cascades to degrade the extracellular matrix and amplify tooth degradation in a dentinal lesion [16]. Tjaderhane *et al*., reported that a slightly acidic resin monomers or adhesives can readily inhibit tissue inhibitor of metalloproteinases-1 in TIMP-MMP complexes leading to the production of active MMPs [17]

Reports cite that the main sources of MMPs in carious tooth are the salivary glands, crevicular and dentinal fluids. MMP-8 and MMP-9 are the most abundant salivary MMPs, which can effectively degrade the exposed dentinal collagen matrix. MMP-2 has been demonstrated in dentinal fluids which are actively secreted by odontoblasts during caries aggravation. MMP-9 and MMP-20 were also been demonstrated in dentinal tubules of carious tooth [18].

## BIOFILMS

Biofilms generally form on varied surfaces including indwelling medical devices and implants, natural and portable piping for water systems, fermentation vessels, food industries, and living tissues [19]. Among bacteria present in biofilm formation, Gram negative bacteria like **Pseudomonas aeruginosa*, Pseudomonas fluorescence, Escherichia coli, Vibrio cholera* being studied for biofilm formation, while *Staphylococcus aureus* and *Staphylococcus epidermis* are the most extensively studied Gram positive bacterial biofilms [20]. Apart from these, **Streptococcus** species like Group A **streptococci*,* Viridans group **streptococci*, Haemophillus influenza,* and *Actinomyces israelli* are also well documented for their biofilm formation in diverse infectious processes. Conversely, bacteria such as **Lactobacillus** species, found in vaginal and intestinal tract flora, forms biofilms which prevent the colonization of harmful pathogens by acting as a protective barrier [21, 22].

### Oral Biofilms and Orodental Infections

One of the preeminent examples of a biofilm that is structurally as well as functionally organized is dental plaque, which is a multi species biofilm comprising of hundreds of bacterial species, salivary polymers, and bacterial extracellular products. The microbial species colonize the teeth, hard palate, tongue, carious lesions, oral mucosa, and periodontal pockets. Supra-gingival and sub-gingival plaques are the most significant biofilms in oral cavity contributing to various orodental manifestations. The distribution of the microbial species in these plaque biofilms varies depending on the anatomical locations and environmental factors [3]. The bacterial plaque adheres resolutely to tooth surfaces as well as restorations and prosthetic appliances. Many molecular and biochemical interactions occur among the bacterial species within biofilm leading to either gradients of carbon dioxide and hydrogen or a strictly anaerobic environment [23].

Of fundamental importance to oral health maintenance and preventative measures to inhibit dental caries, gingivitis, periodontitis, endodontitis, apical periodontitis, and peri-implantitis is the control of oral biofilm formation as these are the major outcomes of oral biofilm [24]. These commensal microbes in the oral cavity function as a principal source of the formation of biofilm in the root canal. The anatomical complexities found within the root canal favor the sustenance and proliferation of biofilm and bacteria. MMPs secreted by the inflammatory cells digests the biofilm components thereby helps to slough of bacterial biofilm. But in a preformed biofilm inside the dentinal tubules and canal, diffusion barrier property to antibiofilm agents, antibacterial drugs and inter canal medicaments like CHX, tetracycline and its derivatives, chemically modified tetracyclines (CMTs) (frequently used chemical MMP inhibitors in dentistry), will enhance MMPs and cysteine cathepsins leading to further tissue destruction [25]. Various surveillance data indicates that approximately 90 percent of carious lesions occur in the pits and fissures of permanent posterior and molar teeth [26]. An increase in biofilm porosity is aided by extracellular polysaccharides enabling penetration of metabolites to the core of biofilm resulting in pH drop [27]. Following this, plaque formation proceeds at an expense of host defenses (saliva inflow rate and frequency and composition of diet) resulting in extensive demineralization of the tooth enamel eventually leading to caries [3]. The inhibition of oral biofilms, as well as the initial microbial colonization, can effectively prevent orodental infections.

### Architecture and Formation of Biofilm

Initiation of biofilm formation begins with the planktonic forms, which are converted into replicating sessile forms on a surface. The cells colonize and irreversibly attach to the surface to be embedded in a self-assembled exopolymeric matrix. Biofilms consist of clusters of bacterial cells, either single species or mixed population. The base unit of biofilm is a cluster or micro colony and is characterized as a discrete group of bacterial cells, from one species or several, enclosed in the matrix [28]. Bacteria and bacterial biofilms are reported in 81.2% of the cementum and root canals as well as 46.8% of the apical delta. When compared to the apical third of the root canal, it was found that a higher bacterial load resides in the cervical and middle thirds [29]. Electron microscopic morphology of biofilms appears as tower or mushroom shaped micro-colony with water channels and interstitial voids within the matrix. These water channels and interstitial voids separate the micro colonies from the external environment through which fluids containing nutrients, waste metabolites, and oxygen move *via* convection (Lawrence *et al.* 1991). Bacteria found at the edges of these fluid channels typically exist in an aerobic environment, while those found within the center of a micro colony are more tightly packed and may live in a strictly anaerobic environment [30].

**Fig. (1):**
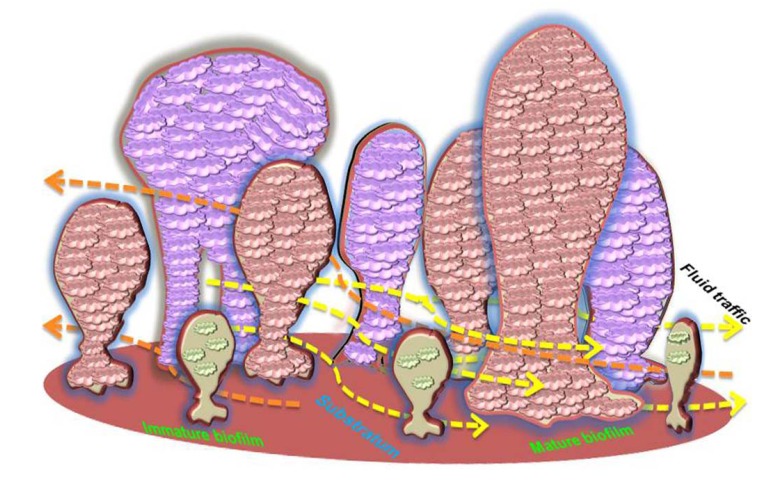
Morphological organization and fluid channeling in biofilm. The schematic representation shows the structure of a mature biofilm showing the mushroom shaped structure adhering to the substratum. The fluid traffic shows the nutrient circulating channels between the micro colonies. The mature biofilm erodes/sloughs off and occasionally forms bridges between the nearest colonies forming a tunnel like morphology.

The assembly and structural organization of such micro colonies can vary depending on bacterial species, the substrate, and the surrounding media. The system of nutrients and metabolic end products distribution functions only in the peripheral regions of biofilms, while differences in oxygen and nutrient availability within the structure of the biofilm can affect the different metabolic activity amongst the cells. In addition, the cells within biofilms secrete signaling molecules serving to activate the formation of micro colonies with complex architecture and diverse functions [31]. This structural complexity of the biofilm provides an effective barrier that limits the penetration of antimicrobial agents throughout the biological layer [32]. The organization of biofilm is displayed in (Fig. **1**).

### Plaque Biofilm: The Precursors to Caries Formation

**Fig. (2):**
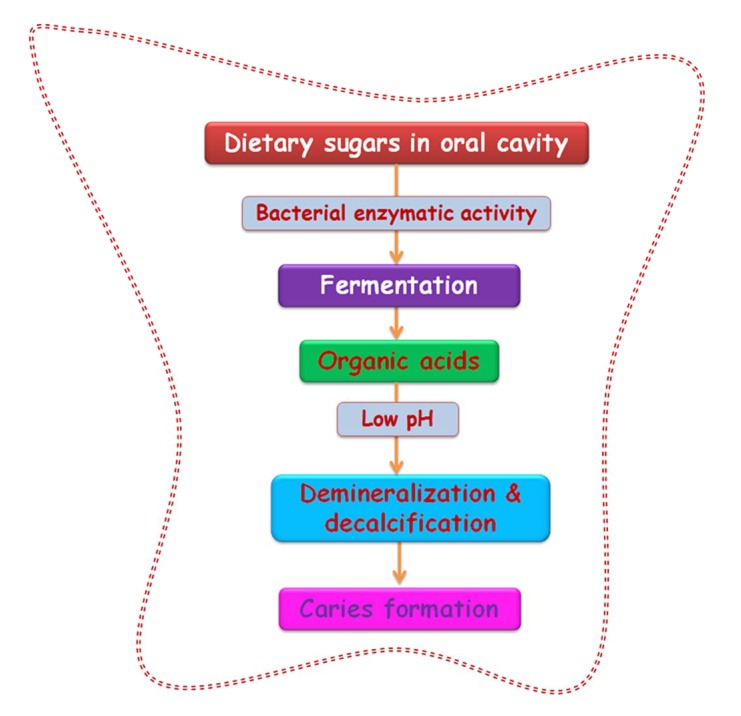
Biochemical events in dental biofilm formation. The fermentation of dietary sugars by the action of bacterial enzymes increase the pool of organic acids and a subsequent drop in pH which in turn activates demineralization and paves way to caries..

**Fig. (3):**
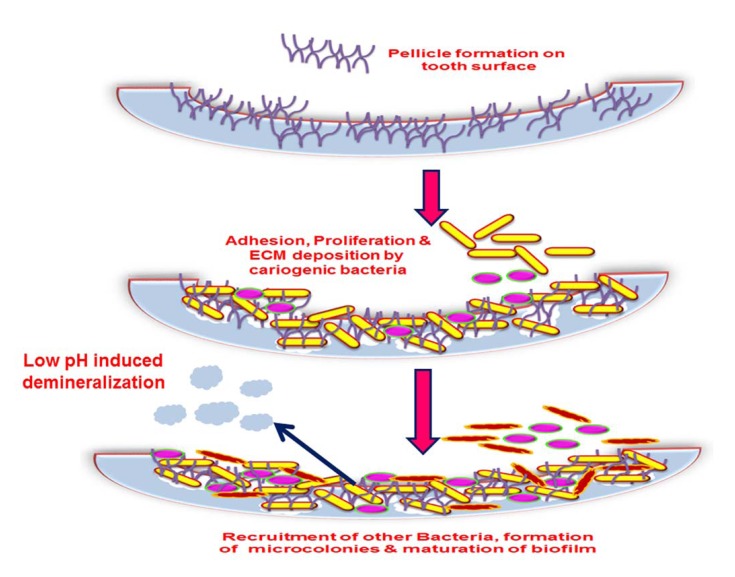
Microbial adhesion and dental plaque formation. Tooth decay is initiated by the pellicle formation at the surface followed by the recruitment, adhesion, proliferation and biofilm deposition. The low pH induced demineralization provides more room for invading bacteria to colonize and eventually leads to decay.

A dental plaque originates by acquired pellicle formation. A pellicle is the salivary component composing of glycoproteins coating, mucins, proline rich proteins, alpha-amylase and other proteins that form on the tooth surface immediately after cleaning. A number of interactions facilitate this bacterial adhesion such as hydrogen bonds, calcium bridges, van der Waals forces, acid-base interactions, hydrophobic interactions and electrostatic interactions which occur between various glycoproteins, salivary components as well as the tooth surface that induce conformational changes to the proteins which form the pellicle [33, 34]. This adhesion to living tissue is mediated through particular molecular components like lectin ligand or adhesins [35]. Result of these interactions is favorable for the colonization of the primary colonizers by a reversible adhesion process aided by the secretion of extracellular polysaccharide matrix (EPS) which assists the bacteria in staying bound together and attaching to the pellicle. The events in biofilm formation are displayed in (Figs. **2** and **3**).

### Key Players of Plaque Biofilm Organization

**Streptococcus* mutans, *Streptococcus* sanguis*, **Streptococcus* oralis, *Streptococcus* gordonii,* and **Lactobacillus* acidophilus* are major pioneer organisms in the plaque formation, which are competitive at the low pH (due to anaerobic metabolism). Oral *streptococci* belongs to four main groups namely *mitis, salivaris, mutans* and *anginosus.* According to Love *et al.,* the streptococcal antigen I/II family of polypeptides mediates the attachment of oral *streptococci* to collagen and determines their ability to invade dentinal tubules found in human teeth. These microbes penetrate the dentinal tubules as co-aggregates as their adaptive response to extreme environmental change makes their survival easy. The pathogenicity of **Streptococcus* anginosus* in root canal is attributable to its attachment and co-aggregation. **Streptococcus* gordonii* recruits *Porphyromonas gingivalis* to dental plaque and promotes its invasion into the dentinal tubules. Biofilm formation has also been shown to be dependent on the intracellular transport of Mn^2+^ in **Streptococcus* gordonii*. While the role of *S. parasanguis* and *S. oralis* in endodontic infections is not known. **Streptococcus* oralis* possesses surface-associated protein which aids in its survival while **Streptococcus* parasanguis* have fimbriae which assist their spreading in the bloodstream and survival during adverse conditions.

**Streptococcus* mutans* shows significant aggregation with **Candida albicans** [36]. **Candida albicans** co-aggregates with **Streptococcus* gordonii, *Streptococcus* sanguis* and other α and non-haemolytic **Streptococcus** species and activates biofilm formation. *Candida* species have various glycoproteins located on the exterior of the cell wall, which help in attachment to the mucosal tissue and abiotic surfaces [37]. *Candida* possesses virulence factors such as adherence, thigmotropism for penetration, hyphae formation, protease secretion and phenotypic switching. They survive in a nutritionally deprived environment via the secretion of aspartyl protease and enzymes which degrade dentinal collagen [38]. Moreover, the *Candida* species can resist the effect of calcium hydroxide during dental restoration therapies. These features of **Candida albicans** indicate their higher rate of survival (10%) within the canal, contributing to root canal and periapical infections [39, 40]. Yeast such as; *Geotrichium candidum, Candida glabrata, Candida krusei, Candida tropicalis, Candida guilliermondii, Candida kefyr and Candida parapsilosis*. are also common in the root canals [41]. They comprise less than 1% of the endodontic flora and are predominantly seen in persistent apical periodontitis [42].

Tahmourespour and Kermanshahi found that **Lactobacillus* acidophilus* causes substantial reduction in the adherence of **Streptococcus* mutans* where the interaction with non-mutan **streptococci** was less significant [43]. Such antagonistic interaction between these species forms the reason for the relative reduced number of mutan **streptococci** in infections of the root canal compared to non-mutans. The acidic environment is advantageous for the cariogens as it inhibits the growth as well as the metabolism of noncariogenic species in biofilm [44]. Other oral microbes like *Actinomyces sp.*, *Veillonella sp.*, *Rothia mucilaginosa, Gemella sp., Capnocytophagae sp., Prevotella sp., Niesseria sp.,* are also frequently found during the early stages of plaque formation [45]. The members of secondary colonizers include *Fusobacterium nucleatum*, *Treponema sp*., *Tannerella forsythensis, Porphyromonas gingivalis, Aggregat-ibacter actinomycetemcomitans* [46]. *Fusobacterium nucleatum* is a well known co-aggregation bridge species that serves as facilitator of *streptococcal* and obligate anaerobes aggregation.

Likewise, ***Enterococcus* faecalis** and **Enterococcus* faecium* harbor in root canals of the carious tooth and elicit persistent asymptomatic infections paving ways to endodontitis [47]. Deng *et al*. reported that the presence of a streptococcal*-*biofilm significantly raises the formation of biofilm in ***Enterococcus* faecalis** strains, but co-aggregation has not yet been reported for ***Enterococcus* faecalis** and **Streptococcus* mutans* [48]. **streptococci** have been isolated in majority of cases with primary endodontic infection, during endodontic treatment and in retreatment, which constitutes approximately 20% of the endodontic mileu in post treatment cases.

Similarly, several ecological factors also regulate the survival of micro-flora found in the root canal. The bacterial synergy in which, the nutritional requirement of one bacterium is met by the metabolic product of the other microbial flora is also responsible for the coexistence of several root canal microorganisms. The majority of the endodontic micro-flora are given in Table (**1**) [49]. Microbial ecology is further influenced by nutritional status found in the coronal parts of the exposed root canal possessing exogenous nutrients (carbohydrates) and the body of the root canal containing endogenous nutrients (proteins and glycoproteins).

### Factors Affecting Biofilm Formation

Proliferation of resident microbes within the biofilm increases the biomass production within the matrix. Various factors including pH, oxygen availability, temperature, and organic metabolites determine the maturation and growth of the biofilm. Moreover, the signaling molecules for cell-cell communication are also integral to population control. During the maturation process of the biofilm, the secondary colonizers or the late colonizers recognize the polysaccharides or protein receptors on the bacterial cell surface that facilitates co-aggregation. These interactions pave the way for the formation of morphologically distinct biofilms like corn cob forms, bristle brush forms, or mesh [68]. Mature biofilms typically contain many porous layers and water channels which provide essential nutrient/metabolite traffic to the residing bacteria [69].

Flushing action of saliva and associated first line of defense, mechanical action of oral cavity during nutrient consumption and fluid sheer force of saliva limit biofilm development. This results in the dispersion of the biofilm cells by erosion, sloughing, and seeding [70]. Also, EPS influences ion exchange within biofilms and controls the hydrophilic/hydrophobic characteristics of biofilm. Oxygen metabolism and exchange of metabolites in the biofilm between the resident aerobes and obligate anaerobes facilitate survival of the latter. Oxygen consumption by the aerobes limits the oxygen content and induces a local reduction potential that provides anaerobic micro-environment for the survival of obligate anaerobes.

The initial attachment of bacteria on to a surface can often result in the upregulation of EPS synthesis. Among the plaque bacteria, mutan **streptococci** and *Lactobacilli*, being acidophilic and aciduric, respectively, have been shown to be capable of demineralizing the dental enamel. Gradually, the bacterial surface structures for adhesion, such as fimbrea, fibrils, and other membrane proteins/glycoproteins, act as cues to interact with the pellicle [71,72]. Biofilm formation by mutan **streptococci**, particularly **Streptococcus* mutans* and **Streptococcus* sorbinus* follows two pathways: sucrose dependent adherence and sucrose independent adherence. In the later route, oral streptococcal surface protein antigen (a cell wall associated adhesin P1 and a member of antigen I/II (AgI/II) encoded by *spaP* gene) is involved [73, 69]. A 29 kDa surface protein known as wall associated protein A (WapA, antigen A or antigen III) also plays a vital structural role at the cell surface for the formation of biofilm and cell to cell interaction [70].

Sucrose dependent adherence is mediated by glucan binding proteins namely GbpA, GbpB, and GbpC of which GbpC was reported to be involved with rapid dextran dependent aggregation [74]. Furthermore, water insoluble glucans produced from sucrose by glucosyl transferase enzymes Gtf-B, Gtf C, and Gtf D also activate microbes for the formation of the biofilm formation as well as acting as a universal glue that forms the plaque matrix [75-78]. Such insoluble extracellular polysaccharides are an essential factor for **Streptococcus* mutans* biofilm formation. However, glucan-binding proteins (GBPs) present within the salivary pellicle mediate initial adherence to the tooth [79]. Reports show that **Streptococcus* mutans* colonizes the dentinal tubule to a greater extend and the depth of penetration is strain dependent [80] and hence also play a significant role in endodontic infections.

Oral **streptococci** produce lactic acid as an end-product of their carbohydrate fermentation process which can lead to a rapid-rate drop in environmental pH. Extracellular pH can modulate hyphal growth of *Candida* and thus biofilm formation as **Candida albicans** grows in the form of yeast at acidic levels of pH and in the hyphal form at alkaline pH levels [81]. For communication among the interspecies to occur between the plaque microbial communities, cell-cell signaling and cell-cell association is required. Co-aggregation is the leading mechanism behind microbial succession by secondary colonizers or late colonizers *via* cell surface adhesins [3]. Cell-cell association may decrease the space between the bacteria and enhance the cell-cell signaling and communication [82]. The polymers found on the surface of cells belonging to the same or different species enables cohesion between the organisms with the rigid alpha-1,3 linked glucans being particularly suited for cohesion [83].

Quorum sensing (QS) controls both intra and inter species communication as well as plays a prominent role in allowing bacteria to architect complex biofilm formation [84]. **Streptococcus* mutans* mediates QS by means of Competence Stimulating Peptides (CSP) and a ComD/ComE two-component signal transduction system. The CSP-mediated QS system found in **Streptococcus* mutans* also affects its aciduricity, acidogenicity, and genetic transformation along with bacteriocin production. Along with QS, autoinducer-2 (AI-2) also favors bacterial interactions [85].

The cell wall specific polysaccharide of **streptococci** and type 2 fimbrea of *Actinomyces* sp. are well reported for highly selective surface interactions [23]. An inducible high-affinity Mn^2+^ transporter in **Streptococcus* gordonii* mediates its co-adhesion with *Actinomyces naeslundii* [23]. A lectin like receptor (ScaA) participates explicitly in the interactions of **streptococci** with *Actinomyces*, while receptors ScbA, PsaA and SsaB resembling the fimbrial adhesins favor co-aggregation [86]. Glucosyl transferase B (GtfB) produced by **Streptococcus* mutans* binds effectively to both hyphal and yeast forms of **Candida albicans** in an enzymatically active form.

 The resultant glucans formed *in situ* enhance the binding ability of **Streptococcus* mutans* cells to the **Candida albicans** cells and promote the colonization of *C. albicans* on the surface of the tooth [87]. Lactobacilli cell surfaces have an S-layer (macromolecular paracrystalline arrays of proteins/glycoproteins) which determines the surface hydrophobicity. The S-layer is thought to act as an active mediator for bacterial adhesion to the host cells/ECM and also have protective and catalytic functions [88]. **Lactobacillus* acidophilus, *Lactobacillus* paracasei, *Lactobacillus* rhamnosus* and **Lactobacillus* plantarum* isolated from the tongue, gums, dental plaque, and the saliva showed high hydrophobic properties [89].

The most potent virulence factors of ***Enterococcus* faecalis** are aggregation substances (AS), enterococcal surface protein (Esp), extracellular superoxide production, the lytic enzymes gelatinase and hyaluronidase, the toxin cytolysins, and capsular polysaccharides. Each of them may be associated with various stages of an endodontic infection and periapical inflammation [90]. It also act as cues for biofilm formation and microbial interactions. Quorum sensing in ***Enterococcus* faecalis** is mediated by auto inducing peptide (AIP) which interacts with the cell surface receptors for co-aggregation and biofilm formation [91].

Multivalent adhesins are present on the cell surface of *Fusobacterium nucleatum* are thus considered as a middle colonizer since they act as a link between the early colonizers and late colonizers. Specifically, an arginine-inhibitable adhesin (RadD) is responsible for its adherence to **Streptococcus** species. Some galactose-specific, lectin-like adhesin are involved in binding to the sugar moiety present within the capsule and lipopolysaccharide of the periodontal pathogens like *Porphyromonas gingivalis*, *Aggregatibacter actinomycetemcomitans* and *Treponema denticola* [83]. *Tannerella forsythia* associated with severe and chronic periodontitis has cell surface glycoproteins in S-layer acting as adhesins in coadhesion with *Fusobacterium nucleatum* [92]. A leucine-rich repeat in proteins from *Treponema denticola* and *Tannerella forsythia* are involved in protein–protein interactions with each other and with *Fusobacterium nucleatum* [93].

Late colonizers have weak colonizing ability and require partner species primarily constituted of anaerobic Gram-negative bacteria to become attached into the dental biofilms [13]. Among this, *Treponema denticola* is unable to adhere on oral surfaces as it is only able to colonize to form oral biofilms when *Porphyromonas gingivalis* is present. Moreover, in sub-gingival dental biofilms, *Treponema denticola* is typically found associated to *Porphyromonas gingivalis* [94]. A chymotrypsin-like proteinase (CTLP) found within a high molecular mass complex on the cell surface of *Treponema denticola* mediates its adherence to other potential periodontal pathogens such as *Porphyromonas gingivalis*, *Fusobacterium nucleatum*, *Prevotella intermedia*, and *Parvimonas micra* [95]. Interaction between *Veillonella atypica* and **Streptococcus** sp. depends on metabolic cooperation provided by their growth in close proximity.

**Fig. (4):**
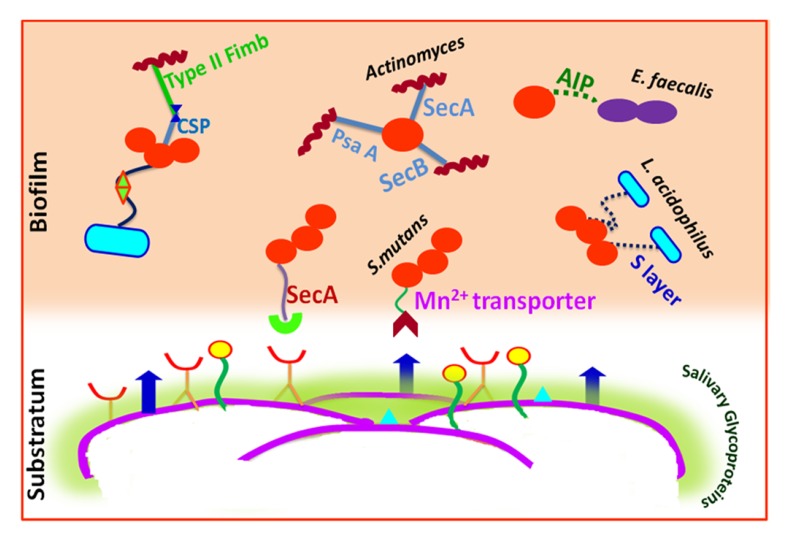
The figure describes well-defined events of initial microbial adhesion to tooth surface favoring biofilm formation. Initial process being the formation of acquired pellicle rich in salivary glycoproteins, mucin and other components on the tooth surface followed by the interaction and adhesion of primary colonizers. Different means of cell-cell interactions among the microbes are also depicted.

Metabolite mediated cooperation also favors physiological interactions among the biofilm bacteria. Metabolic communications among oral microbes may occur through the excretion of a metabolite by one bacterium that nourishes another species [96] or through syntrophic biochemical enzymes to cooperatively metabolize a substrate. For instance, the growth of **Streptococcus* sp.* leads to production of lactic acid which acts as a substrate for growth of *Veillonella sp*. [97]. Also, the short-chain acids like acetate that are produced by the early colonizers *via* sugar metabolism typically serve as sources of carbon and energy for succeeding colonizers [83]. Interactions occurring among the major cariogens that lead to biofilm formation are shown in (Fig. **4**).

Dental caries and periodontitis are the two most important biofilm mediated oral infections. The progression and prevention of dental caries can be arrested by daily habits and clinical therapies for promoting the remineralization. The replacement of lost hydroxyapatite of the tooth tissue by fluorides or other ions and prevention of oral biofilm formation that acts to reduce the pH are mostly employed. Measures of biofilm control like mechanical removal of plaque, systematic and local antibiotics or interventions that artificially increase saliva production are mostly preferred. A prominent reason of primary and secondary endodontic infection can be attributed to the ***Enterococcus* faecalis** biofilms. The widespread occurrence of ***Enterococcus* faecalis** in primary endodontic infection has been reported to be 40% and in persistent endodontic infection 24 to 77%. ***Enterococcus* faecalis**, by virtue of its ability to survive severe environments including detergents, azide, heavy metals, desiccation, and ethanol, colonizes the dentinal tubules and other inaccessible areas within the oral cavity. It also survives high temperature, alkaline pH, salt concentration and bile salts. The biofilm forming ability enables ***Enterococcus* faecalis** to survive phagocytosis, antimicrobials and antibodies (Mohamed and Huang, 2007). The inter canal medicaments and dressings even though offer solution, but its failure in inhibiting such microbes and biofilm results in failure of typical root canal therapies. Though canal cannot be reliably rendered free of all bacteria in all of the cases, applying a temporary filling material with an inherent or incorporated microbicidal agent may reduce the overall microbial load.

## SECONDARY CARIES

Secondary caries is similar to primary dental caries which are accelerated by the activity of the microbial population within the dental plaque, mainly in proximity of a restoration [98]. In primary caries, as the tooth mineral is lost, the proteinases of plaque bacteria cause the secondary destruction of tooth protein leading to cavitation. On the other hand, in secondary caries the bacteria may come from oral cavity itself to get trapped into anaerobic environment of the micro-cracks along the tooth-restoration interface. This suggests that the first step and contribution to cavity formation may be the destruction of the tooth protein. This is then followed by fermentation of present dietary carbohydrates that accumulate and produce more acids leading to lesion [99]. Reports indicate that over half of operative dentistry performed on adults involves the replacement of restorations based on the diagnosis of secondary caries [98].

Secondary caries can appear as a wall lesion or as a superficial lesion adjacent to a restoration [100]. The gingival surfaces are more susceptible to secondary caries due to the inability to maintain plaque free conditions mainly in inter-proximal regions. The gingival surface is also prone to contamination by gingival fluid and saliva. Moreover, the improper insertion of restorative materials also aggravates the situation. Also, less effective bonding of the resin composite and polymerization shrinkage at gingival cavo-surface influences the integrity of restoration [98]. Mertz-Fairhurst *et al.* demonstrated that active caries may become inactive or arrested when adequately sealed from the oral environment [101]. Silva *et al.* reported that biofilm accumulation is typically observed at the tooth surface interface or overhangs on Class II amalgam restorations [102]. The rougher restorations, due to faulty cavity preparation, insertion, carving, and finishing, could also favor dental biofilm retention. These are the decisive factors for secondary caries [103].

Micro-leakage occurs when micro cracks (more than 50 micrometer diameter) facilitate the seepage of saliva, which drains the bacterial cells into the treated tooth. These cracks also act as an ecological niche for the growth of anaerobes [99]. A recent study reported that micro-leakage and associated secondary caries forms the reason for the failure of a large percentage of dental restoration [104].

The use of amalgams strengthened with fluoride, functional monomers such as 10-methacryloyloxyethyl dihydrogen phosphate, and dental composites that act as acid-base resistant zone (ABRZ) were highly appreciated [105] as they form a barrier between the adhesive layer and the tooth. A handful of contrasting literature reports indicate that fluoride-releasing restorative materials have not shown to be very successful due to a lack of sustained antimicrobial activity [98].

### Microbial Etiology of Secondary Caries

The microorganisms occupying the root canal and various endodontic regions were well established [106]. Bacteria present in dental pulp are associated with periapical diseases. The chemo-mechanical preparation of the tooth during the root canal treatment helps in the removal of the bacterial flora, but the failure in complete removal of the bacterial cells during the treatment or entry of the bacterial cells and nutrients into the root canal *via* micro-leakage can be a reason for residual caries or secondary caries [107]. Still, microbiology of the secondary caries has not received much attention and also is a disputed matter [108].

Gonzalez-Cabezas elucidated the active roles of **Streptococcus* mutans, Lactobacilli,* and *Actinomyces naeslundii* in the development of secondary caries [87]. Mo *et al.* explored the most predominant micro-flora of secondary caries with the major candidates including **streptococci* mutans, Lactobacilli, Prevotella, Veillonella*, *Neisseriae* followed by *Actinomyces, Pepto*Streptococcus*, Fusobacterium, Porphyromonas gingivalis,* and occasionally *Capnocytophaga* [99]. *Actinomyces odontolyticus* and *Candida spp*. were also reported to be key players [109]. The presence of obligate anaerobic species and facultative anaerobes in the secondary caries suggests a bacterial spectrum similar to the micro-flora of subgingival plaque associated with periodontal disease and pulp pathogens. Gonzalea-cabezas also reported the distribution of three cariogenic bacteria: mutans *streptococci*, *Actinomyces naeslundii*, and **Lactobacillus* sp*. Among these, 88.9% were mutan *streptococci* in secondary carious lesions occurring around amalgam restorations [110].

Residual caries persists because of sustenance and viability of primary infection throughout the treatment procedure as well as failure of the endodontic irrigant to eliminate bacteria or the result of an inability of chemo-mechanical instrumentation due to survival of bacteria in various inaccessible locations (isthmuses, accessory canal and apical regions of canals) [111]. Zivko *et al.* identified undetected residual caries under old fillings that led to the failure of prosthetic dental treatment [112]. Nair *et al*., reported the difficulties to differentiate between the microorganisms existing from primary infections and the ones that contribute to the secondary infection [111]. 

It is to be noted that the dentin organic materials contain inherent proteases particularly MMPs and cysteine cathepsins which are activated in the acidic environment. This would further degrade the treated tooth. It is already proven that MMP inhibition controls dentin caries progression and allows natural remineralization of the treated tooth. Hence, during the treatment of coronal caries (mainly in deep and active caries lesions) direct application of MMP solution is performed after the mechanical removal of carious residues before restoration. Chlorhexidine digluconate (CHX) is frequently used MMP inhibitor in dentistry acts by calcium-chelating mechanism but its long term affectivity in a treated tooth is not sure [113, 114]. These inhibitors have been shown to improve the integrity of the hybrid layers obtained by a simplified etch-and-rinse adhesive after dentin caries removal. Another indicative is to incorporate in mouthwashes and toothpastes. In periodontal infections oral administration of MMP inhibitors are also practiced [15]. The acidic activation elevates the level of MMP-2 and MMP-9 which could be due to the inhibition of TIMPs. This could also contribute to further degradation of endodontically treated tooth and aggravate the residual caries [115].

## TREATMENT MODALITIES FOR PRIMARY AND SECONDARY CARIES

In the beginning of twentieth century, the main treatment modality was simply the debridement and restoration without addressing the real cause of dental caries. The failure of these treatments paved way to the methods of either tooth extraction or radical removal of diseased portion along with material-driven geometric extensions to the caries resistant areas. This concept was introduced by G V Black and is known as ‘Extension for Prevention’ which has prevailed in dentistry for more than 120 years [116]. By the second half of twentieth century, more emphasis was given to patient education regarding preventive measures [116]. Extensive research in cariology led to the understanding of the root cause of disease and its progression. Hence, the changes in concept of different treatment modalities were also emphasized and investigated. Resin-based photo sealant for pits and fissures over dentin in non-cavitated caries lesions can significantly reduce caries progression, but has the disadvantage of instability [117]. Various investigations supported that the caries progression can be controlled by restricting the amount of nutrients used for bacterial metabolism within the lesion through the cavity sealing. Once acid production by the microbial metabolism is controlled, caries process can also be controlled, regardless of bacteria found in the dental tissue [101].

In restorative dental practices, amalgam and composite restorations are used most often. Over time, these restorations have to be replaced for various reasons including the development of secondary caries, fracture of restorations, and overflowing fillings. These restorations are required to be checked regularly and, when necessary, repaired or replaced [104]. However, after each filling is replaced, the cavity is unavoidably widened by a mean of 0.6mm. Minimally invasive dentistry is designed to promote the maximum preservation of healthy dental structures by the removal of soft demineralized dentine and underlying infected tissue before placing a filling or a restoration. The removal is aided with excavators in an effort to avoid further development of the decay. Such drilling and filling type of treatment has been widely accepted and practiced for generations by dentists [118]. This approach has been challenged by three different methods: (1) sealing the tooth decay without removing the caries [119], (2) removal of minimal caries at the entrance to a cavity and sealing the cavity by retaining the remaining caries [101], and (3) step-wise removal of the caries by employing excavation technique. The later approach involves ultra-conservative removal of the caries and the placement of temporary restorative material. This temporary filler is removed after a few weeks as well as all the remaining decay which is then replaced with a permanent filling [120]. The minimally invasive treatment modalities are much preferred to reduce excessive sacrifice of the tooth tissues in spite of its constraints and failure [121].

The prevention and preservation approach is significant as the caries development is a slow process. Hence, preventing early carious lesions by the removal of biofilm as well as the application of fluoride or placement of sealants is advised [122]. The main flaws of this approach are the short durability of restorations and the propensity of new caries at the margins of restorations if not cured properly [98]. When carious enamel is retained at the margin of the cavity, the ingress of plaque biofilm bacteria through the micro-pores *via* defective enamel structure occurs and leads to cohesive micro-leakages. Further complications are the chances of secondary caries along defective marginal interfaces and failure of the restorations [123].

The primary rationale for endodontic treatment is to eradicate the infection by reducing the load of bacteria. The next is to create an unfavorable environment for the survival of cariogenic bacteria. This prevents the microorganisms from infecting or re-infecting the root and/or the peri-radicular tissues. In an effort to remove the organic and inorganic debris, smear layers, and bacteria from the entire root canal system, as well as maintain dentine permeability, combinations of aseptic treatment techniques, chemo-mechanical preparation of the root canal, intra canal medicaments, and antimicrobial irrigating solutions are required. However, several studies have reported that the chemo-mechanical preparation clears only about 50-70% of infected canals and microorganisms since the efficiency is dependent on the antimicrobial irrigation regimen and complexity of the canal [124].

Residual pulpal tissue, bacteria, and dentine debris may remain within the irregularities of root canal systems even after thorough mechanical preparation. Therefore, an irrigant solution should be implemented along with canal preparation. Root canal irrigants are used as antimicrobial agents as well as a way to flush out loose debris, lubricate the dentinal walls, and dissolve organic compounds in the root canal. Commonly used irrigants include sodium hypochlorite (NaOCl), citric acid, ethylene diamine tetra-acetic acid (EDTA), chlorhexidine (CHX), and a mixture of tetracycline, an acid, and a detergent (MTAD). The high pH of NaOCl interferes with cytoplasmatic membrane integrity, causing irreversible enzymatic inhibition, which induces biosynthetic alterations in bacterial metabolism and phospholipids destruction. The ability to dissolve organic substances remaining in the root canal system and the low cost makes NaOCl an optimal irrigant for dental applications. When injected in periradicular tissues, the cytotoxicity, foul smell and taste, ability to bleach and corrosion of metal objects form hurdles in using NaOCl. Moreover, it was found that 2% CHX and 2% NaOCl was only able to kill 13% - 15% of 3-week-old biofilm bacteria within dentin after one minute of exposure [125]. The application of MMP inhibitors which inhibits the breakdown of dentin collagen within the hybrid layers can also to prevent the occurrence of secondary caries around restorations [114].

The complete eradication of bacteria in the root canal during a single treatment session is still a challenge. Therefore, an efficient antimicrobial agent is required to remove the bacteria residing on the un-accessed sites of the root canal. In addition, the antimicrobial agents used as short-term inter-canal medicament must have the ability to penetrate through dental tissues in the presence of microbes and retain its microbicidal activity. The deleterious effects of such synthetic chemicals on teeth and oral tissue have prompted an investigation for efficient herbal drugs which have lesser side effects.

## ANTICARIOGENIC PHYTOTHERAPY

The use of plants for dental applications is very pronounced in the history of dental sciences and phytodentistry is gaining its appreciation in current scenario. Several plant parts have been implemented for tooth cleaning purpose for thousands of years, even before ancient Babylonian civilizations (5000 BC) [126]. The bioactive properties including anti-inflammation, antiseptic, antiedema, analgesic, healing, lesser side effects, *etc*. make phytomedicine an ideal therapeutic strategy for oral problems [127]. The conventional chemical drugs used for the management of dental caries may evoke undesirable effects like vomiting, diarrhea, and staining of the tooth, emergence of drug resistance [128, 129]. The hunt for alternative chemicals with lessened side effects ended in phytochemicals from different sources. Such phytomoieties should be antibacterial and their ability to prevent inflammation allergy, oxidative stress, and carcinogenesis provide added benefits. Crude and purified extracts of several plants have been shown to be effective against a series of oral pathogens.

Wide varieties of antimicrobial plant products are being used in dentistry especially as components in toothpastes and mouth rinsing solutions. These phytochemicals supplements are very effective in preventing the caries and associated biofilms [130]. Parodontax® (GlaxoSmithKline, Middlesex, UK), a widely used tooth paste, contains the extracts from *Matricaria chamomilla, Echinacea purpurea*, *Salvia officinalis, Commiphora myrrha,* and *Mentha piperita* which provide anti-inflammatory, immune stimulant, antihemorrhagic, antiseptic, and analgesic properties, respectively [131]. Moreover, the introduction of these plant extracts to a mouth rinse has significantly reduced the gingival index (a measure of periodontal disease based on severity and location of the lesion) [132]. *Aloe vera* extracts have been reported as effective against aphthous ulcers and alveolar osteitis owing to its anti-inflammatory and antibacterial effects [133]. Extracts from *Vernonia amygdalina, Fagara zanthoxyloides, and Massularia acuminata* were reported to be potent against the oral anaerobes like *Porphyromonas gingivalis, Prevotella intermedia, Fusobacterium nucleatum, Eikenella corrodens,* and *Campylobacter rectus* [134]. The common phytochemicals with anticariogenic activities are given in (Fig. **5**).

The combination therapy of *Centella asiatica* and *Punica granatum* exhibited significant reduction in periodontitis indexes mediated through IL-1β and IL-6 [135]. *Salvadora persica* inhibited the growth as well as biofilm formation of **Enterococcus* fecalis* and *Aggregatibacter actinomycetemcomitans* and prevented associated dental plaque formation and was found to be more effective than 0.2% CHX [136, 137]. The inhibitory activities of *Azadirachta indica, Mikania laevigata, Allium cepa*, *Allium sativum* and *Mikania glomerata* against different strains of **Streptococcus* mutans* and other oral pathogens, revealed their efficiency to lower dental caries and associated lesions [138-140]. Tea (*Camellia sinensis*), possesses abundant antibacterial phytochemicals and was effective against **Streptococcus* salivarius* and **Streptococcus* mutans* [141]. Inhibition of salivary amylase enzyme by tea born phytochemicals is beneficial for reducing carbohydrate induced dental caries [142, 143]. Owing to its growth inhibition against ***Enterococcus* faecalis**, *Hyptis divaricate* has also been considered to be effective alternative for intracanal medicament [144]. Oral bacteria like *Fusobacterium nucleatum*, *Prevotella nigrescens*, and *Bacteroides fragilis* were arrested by the castor oil from *Ricinnus communis* [145]. Also, the ricinoleic acid induced inhibition of bacterial proliferation on hydroxyapatite surfaces has already been reported [146].

Roots and stolon of *Glycyrrhiza glabra,* has been hailed for its pharmacological properties against viral and bacterial infection, inflammation, oxidative stress, oral ulcers, dental decay, and cancer. These pharmacological effects can be attributed to the abundance of triterpene, saponin and glycyrrhizin (chemically diglucuronide of glycyrrhetinic acid). Its non-sugar sweetening property is being exploited in various pharmaceutical preparations [147]. Among these phytochemicals glycyrrhizin is well known for its anticariogenic effects owing to its antibacterial and biofilm inhibitory effects. The anti-inflammatory action of glycyrrhizin mediated through the inhibition of phospholipase A2 and cyclooxygenase activity has added extra incentive for the use of G*lycyrrhiza glabra* for dental therapeutics [148].

Several plants have been hailed to be the excellent source of natural exogenous inhibitors of MMPs which have better therapeutic potential with limited side effects. Kato et al., has reported that epigallocatechin gallate (EGCG), a green tea polyphenol, has potent inhibitory activity against MT1-MMP, resulting in the decreased MMP-2 activation. Also, EGCG directly inhibits MMP-2 and MMP-9 and inhibits dentinal erosion [149]. Similarly, grape seed extract (GSE) suppress lipopolysaccharide-induced MMP secretion by macrophages and thereby inhibiting MMP-1 and MMP-9 activity in periodontitis. The seeds of *Lupinus albus* are also effectively decrease the expression of MMP-9 and MMP-2 by gingival fibroblasts in periodontal infections [150]. The MMP-inhibitory effects of cranberry proanthocyanidins also suggest that they could be effective in treating down dentin caries progression [15]. Extracts of Soya bean unsaponifiables, avocado and oleic acid are also known for their effective MMP- inhibition *in *vitro** [114].

In a polymicrobial environment like that of an oral cavity, phytotherapeutic agent with multiple mode of action may offer promising outcomes. Likewise, the phyto-agent with broad spectrum of activity against both aerobic and anaerobic oral pathogens along with excellent antioxidant, anti-inflammatory and MMPs and other proteolytic enzyme inhibitory activity can be an ideal choice. These bioactive properties enhance healing and regeneration of the affected tissues in the oral cavity adjunct with the antimicrobial activity. Moreover, the phyto-constituents with an ability to inhibit initial adherence of the organisms onto the tooth surface along with anti-biofilm activity and slime layer removal ability can be an ideal agent for oral application. Furthermore, the same phytoextract with the ability to inhibit the host etiological agents favoring the caries and other oral diseases will be an added advantage in dental therapeutics. The combination phytotherapy can also be promising where the selection of relevant phytochemicals and understanding their mode of action are crucial (Table **2**).

## SUMMARY AND FUTURE DIRECTIONS

Understanding the basic mechanisms leading to dental plaque and carious lesions are the key to develop an appropriate therapeutic strategy. The side effects and inefficiency of current management approaches led to the hunt for alternative, personalized and precision medicine. The bioactive, medicinal and therapeutic properties of phytochemicals are promising in phytodentistry. Phytoagents, owing to their multiple mode of action can efficiently minimize the side effects, toxicity and other undesirable effects of the current dental therapeutic agents. But the major challenges in phytodentistry like bioavailability and bioretention of the phytoagents demands for continuous administration as well as higher dosage. The site-specific delivery of phytochemicals can be appreciated for enhancing the efficiency of plant based dental therapeutics. This can be achieved by biocompatible polymer based scaffold loaded with the phytochemical to facilitate a continuous release of the therapeutic compound. Hence, the incorporation of a drug loaded polymeric hydrogel scaffold or a hydrogel biocomposite inside the prepared cavity might help to prevent secondary as well as residual caries may and adjuncts the Atraumatic restorative treatment (ART) approach. Minimally invasive (MI) dental practices dealing with the selective removal of heavily infected and irreversibly denatured dentine caused by carious lesions by preserving dentine can be much benefited with such approaches. But the conventional excavation methods based on the color and hardness of dentine clinically presents difficulty to detect the boundary leading to either under or over excavation. The incorporation of a remineralizing agent like hydroxyapatite or a buffering agent to neutralize the acid production, in the hydrogel scaffold along with the antimicrobial agent would further assist in success of minimally invasive dentistry. This could increase the remineralization potential along with inhibition of further progression of the carious lesion. The incorporation of phytochemicals with extensive therapeutic potential to the conventional as well as advanced dental practices can open new opportunities for millions of sufferers throughout the globe.

## Figures and Tables

**Fig. (5a):**
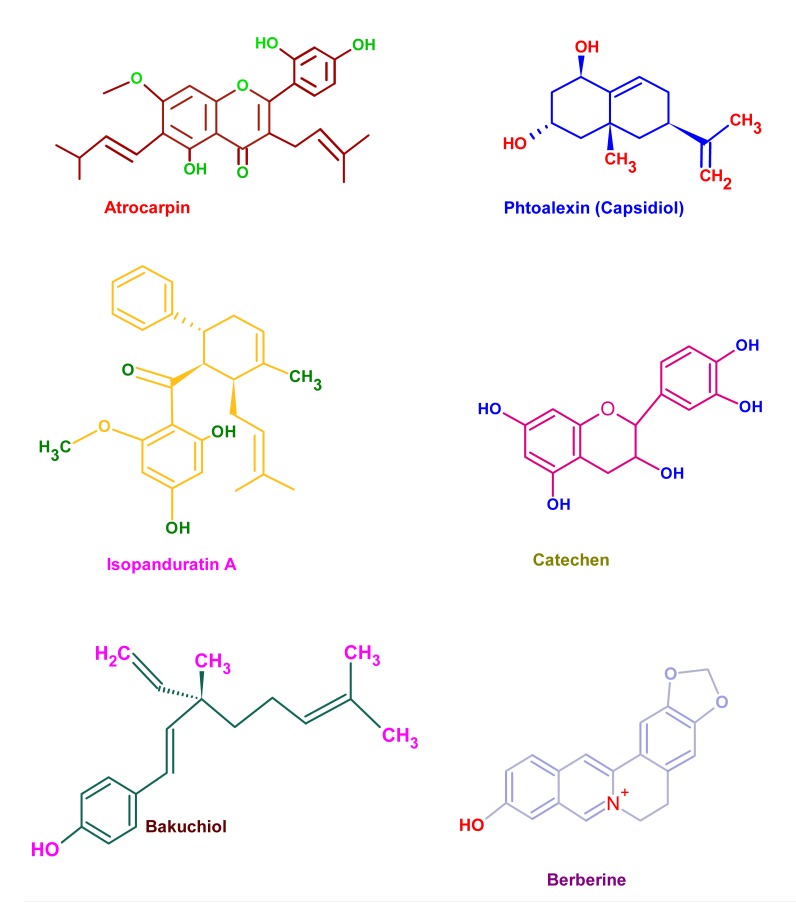
Figs. **A and B**. Structure of phytochemicals having anti-cariogenic properties. The active phyto-components possess unique side chains with highly reactive but stable functional groups which imparts their biological function. These moieties or their metabolites can bind to cellular or extra cellular components to elicit their biological function.

**Fig. (5b):**
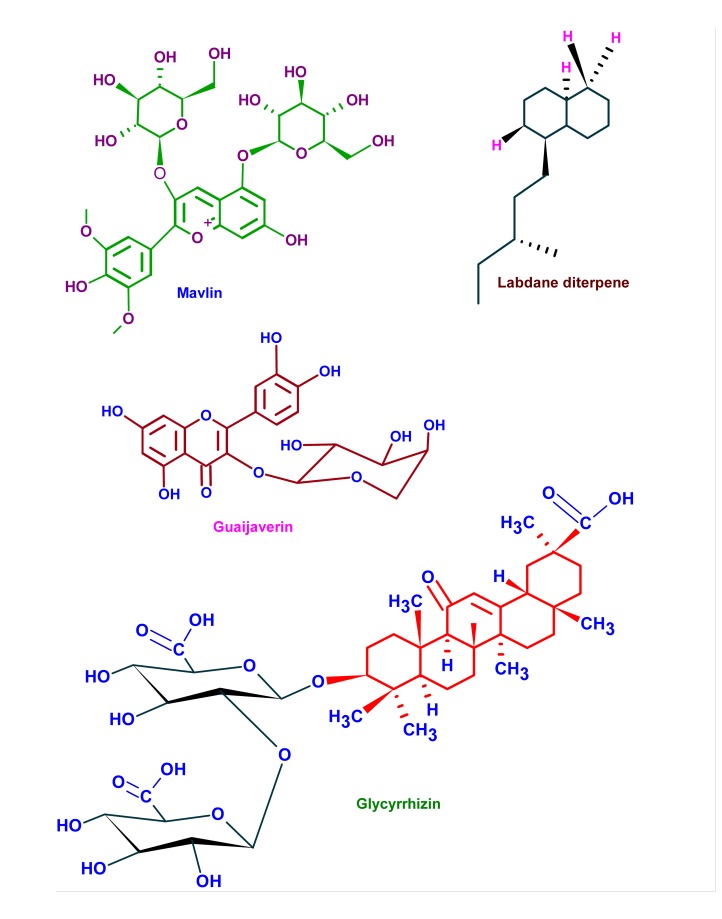
Figs. **A and B**. Structure of phytochemicals having anti-cariogenic properties. The active phyto-components possess unique side chains with highly reactive but stable functional groups which imparts their biological function. These moieties or their metabolites can bind to cellular or extra cellular components to elicit their biological function.

**Table 1:** Common contributor microbes for endodontic biofilm.

**Species**	**Reference**	**Species**	**Reference**
*Porphyromonas asaccaharolytica*	[50]	*Bacteroides fragilis*	[51]
*Prevotella melaninogenica*	[52]	**Eubacterium* limosum*	[50]
*Prevotella bivia*	[52]	**Eubacterium* aerofaciens*	[53]
*Prevotella oulora*	[54]	*Fusobacterium nucleatum*	[55]
*Tissierella praeacuta*	[51]	*Fusobacterium* canifelinum	[56]
***Enterococcus* faecalis**	[57]	*Fusobacterium naviforme*	[52]
*Bifidobacterium adolescentis*	[58]	***Lactobacillus* reuteri**	[59]
*Clostridium histolyticum*	[57]	**Lactobacillus* casei*	[60]
*Pepto*Streptococcus* productus*	[61]	**Lactobacillus* alactosus*	[60]
*Pideococcus parvulus*	[62]	**Lactobacillus* fermentum*	[60]
*Pepto*Streptococcus* asaccharolyticus*	[63]	**Lactobacillus* plantarum*	[60]
*Pepto*Streptococcus* magnus*	[64]	*Prevotella dentalis*	[52]
**Eubacterium* tenue*	[65]	*Enterobacter agglomerans*	[66]
**Eubacterium* infirmum*	[67]	*Staphylococcus epidermidis*	[57]
**Eubacterium* saburreum*	[67]	**Streptococcus* aureus*	[57]

**Table 2:** Common anti-cariogenic phytochemicals and their sources.

**Phytochemical**	**Plant**
Artocarpin	*Artocarpus heterophyllus*
Artocarpesin	*Artocarpus heterophyllus*
Phytoalexins	*Sophora exigua*
Isopanduratin A	*Kaempferia pandurate*
Catechins	*Camelia sinensis*
Malvin	*Alcea longipedicellata*
Bakuchiol	*Psoralea corylifolia*
Berberine	*Coptidis rhizoma*
Guaijaverin	*Psidium guajava*
Labdane diterpene	*Sagittaria sagittifolia*
